# An index of left ventricular diastolic dysfunction in patients starting peritoneal dialysis might indicate mortality and peritoneal dialysis discontinuation

**DOI:** 10.1007/s10157-026-02913-x

**Published:** 2026-07-11

**Authors:** Yoshifumi Hamasaki, Marie Morota, Masaomi Nangaku

**Affiliations:** 1 Department of Hemodialysis and Apheresis, The University of Tokyo Hospital, Tokyo, Japan; 2 Department of Nephrology and Endocrinology, The University of Tokyo Hospital, Tokyo, Japan

**Keywords:** E/e′, Left ventricular diastolic dysfunction, Peritoneal dialysis, Mortality, Peritoneal dialysis discontinuation, Tissue Doppler echocardiography

## Abstract

**Background:**

The E/e′ ratio is recognized as a reliable and sensitive echocardiographic marker of left ventricular diastolic dysfunction (LVDD). Increased E/e′ is known to be associated with mortality and cardiovascular events in kidney failure patients undergoing maintenance replacement therapy. To elucidate the prognostic significance of E/e′ in patients starting peritoneal dialysis (PD), we investigated the relations between baseline E/e′ after PD initiation and death or PD discontinuation.

**Methods:**

Data were collected retrospectively from patients with the E/e′ ratio evaluated early after PD initiation during August 2008 through December 2023. We investigated the association between the average of septal and lateral E/e′ (E/e′ Avg) and a composite outcome comprising mortality and PD discontinuation within 8 years after PD initiation.

**Results:**

This study included 74 incident PD patients with median age of 64 years (74% male). The median value of E/e′ Avg was 9.4 at the median time of 3.3 months after PD initiation. Based on the cut-off value inferred from receiver operating characteristic analysis, patients were divided into two groups: patients with E/e′ Avg ≤ 9.0 and with E/e′ Avg > 9.0. Kaplan–Meier analysis indicated the 8-year survival and PD continuation rates as significantly lower for the E/e′ Avg > 9.0 group than for the E/e′ Avg ≤ 9.0 group (*p* = .0019). Cox proportional hazards analyses showed E/e′ Avg as independently associated with the composite outcome (hazard ratio 1.17, 95% confidence interval 1.07–1.28).

**Conclusion:**

Increased E/e′ Avg early after PD initiation is associated positively with 8-year mortality and PD discontinuation.

**Supplementary Information:**

The online version contains supplementary material available at https://doi.org/10.1007/s10157-026-02913-x.

## Introduction

Peritoneal dialysis (PD), a home-based treatment option of kidney replacement therapies (KRT) for patients with kidney failure, is administered to 11% of all dialysis patients worldwide [1]. Compared to hemodialysis (HD), PD presents several important benefits such as better quality of life, less need for vascular access, and better preservation of residual kidney function. However, as in HD patients, cardiovascular disease (CVD) is the leading cause of mortality, accounting for more than half of all deaths of PD patients [1]. In addition, discontinuation of PD and transfer to HD based on various causes such as PD-related infection and fluid overload is an important issue to be addressed [1, 2].

Left ventricular diastolic dysfunction (LVDD), defined as an impairment of active relaxation and passive elastic properties of the left ventricle as well as a resulting increase in LV filling pressure, is a cardiac functional abnormality associated strongly with the symptoms and severity of heart failure [3, 4]. Irrespective of the presence of symptoms, LVDD is common, with prevalence of 30–75% among kidney failure patients undergoing replacement therapy [5, 6]. The severity of LVDD is independently associated with prognosis, not only in the general population but also in patients undergoing HD or PD, even when left ventricular systolic function is preserved [7–10]. Reportedly, LVDD occurs earlier than systolic dysfunction; it is closely associated with symptoms of heart failure [4, 11]. Therefore, early detection and evaluation of LVDD are expected to be crucially important for stratifying risks of adverse outcomes and for planning appropriate treatment and care for patients with kidney failure.

Diagnosis and severity assessment of LVDD are generally performed using non-invasive examination: echocardiography. Among echocardiographic parameters related to left ventricular diastolic function, the E/e′ ratio is recognized as a reliable and sensitive marker for estimating left ventricular filling pressure, and also as a diagnostic criterion for LVDD [3, 4, 7, 12, 13]. The E/e′ ratio is calculated using both transmitral LV inflow velocity pattern data from pulsed Doppler and peak early diastolic mitral annulus velocity values from tissue Doppler imaging (TDI). Recent recommendations and guidelines have proposed a value of E/e′ exceeding 14 as one diagnostic criterion for LVDD [3, 4]. Earlier reports have described that increased E/e′ constitutes an independent risk factor associated with mortality, cardiovascular (CV) events, and decline of residual kidney function in patients on maintenance HD or PD [6–8, 12, 14]. Because E/e′ increases with dialysis vintage, early evaluation after initiation of PD is desirable [6, 15].

Although significant correlation between increased E/e′ and adverse outcomes has been demonstrated in maintenance HD and in PD patients, few reports have described studies of the relation between E/e′ evaluated soon after PD initiation and long-term prognosis. The present study was conducted to elucidate the relations between baseline E/e′ and outcomes consisting of mortality and PD discontinuation within eight years in incident PD patients with preserved left ventricular systolic function.

## Materials and methods

### Study design and patients

We conducted a retrospective observational cohort study including patients initiating PD between August 2008 and December 2023 at a tertiary care hospital. Inclusion criteria were the following: (1) age of 18 years or older when starting PD, (2) patients initiating PD as their first maintenance kidney replacement therapy, and (3) patients inducted into PD under inpatient care according to our usual clinical practice policy, with continued follow-up at an outpatient clinic department at our hospital. All patients were treated using low GDP and neutral pH glucose-containing PD solutions. Exclusion criteria were the following: (1) patients with no E/e′ data within 6 months after PD initiation, (2) conditions under which E/e′ cannot be applied for LVDD evaluation [post heart transplantation (*N* = 1) and pacing (*N* = 2)], (3) reduced left ventricular ejection function (LVEF less than 50%), and (4) patients who declined to participate in this study. Written informed consent was waived because of the characteristics of the retrospective observational study, but opt-out consent was adopted. We provided participants with both information about the study (purpose, method, required data, and duration) on a website and the opportunity for opting out. This study, conducted in accordance with the principles outlined in the Declaration of Helsinki, was approved by the Research Ethics Committee of the Faculty of Medicine of the University of Tokyo (approval number #2269) on December 22, 2008.

### Data collection

Basic participant information was obtained from participants’ electronic medical records: age, gender, cause of kidney failure, and history of CVD before PD initiation. Data of body mass index, systolic and diastolic blood pressure, use of angiotensin-converting enzyme inhibitors (ACE-i) or angiotensin receptor blockers (ARB), use of beta-blockers, use of automated PD devices, use of 2.5% glucose and icodextrin-based PD solutions, total and renal weekly Kt/V, and the dialysate-to-plasma creatinine concentration ratio at 4 h (D/P Cr) as evaluated by peritoneal equilibration test (PET) were obtained from electronic medical records. Laboratory data were also obtained from electronic medical records on the day of first PET after PD initiation: hemoglobin (Hb), albumin (Alb), blood urea nitrogen (BUN), serum creatinine (Cr), corrected calcium, phosphate, C-reactive protein (CRP), and brain natriuretic peptide (BNP).

### Echocardiographic measurement

Examination by transthoracic echocardiography was performed within 6 months after PD initiation as part of our routine medical care provided to patients. All echocardiographic examinations were performed by an experienced specialist using commercially available equipment. Cardiologists confirmed the obtained measurement data. The two-dimensional and M-mode echocardiograms were used to measure the chamber size, wall thickness, and LVEF. The data of E/A, the ratio between the early diastolic transmitral flow velocity (E wave) to the late diastolic velocity caused by atrial contraction (A wave) as measured using pulsed Doppler echocardiography, were obtained from electrical medical records. Each septal and lateral E/e′ ratio was calculated using transmitral LV inflow velocity pattern data from pulsed Doppler and peak early diastolic mitral annulus velocity values from TDI as follows: E/e′ = [Peak velocity of the early diastolic wave (E)]/[Peak early diastolic mitral annulus velocity (e′)] [3, 4]. Based on the data of septal and lateral E/e′ recorded in the electrical medical records, we calculated the mean values of septal and lateral E/e′ ratios (E/e′ Avg). Left ventricular hypertrophy (LVH) was defined as a left ventricular mass index (LVMI) exceeding 115 g/m^2^ in a male patient and 95 g/m^2^ in a female patient, as reported earlier in the literature [16, 17].

### Outcomes

The primary outcome was the composite of all-cause death and PD discontinuation within 8 years after PD initiation. PD discontinuation was defined as the complete transfer to HD. Switch from PD monotherapy to combined therapy with PD and once-weekly HD was regarded as continuation of PD therapy. Data of occurrence of the first outcome and the date of the outcome for each patient after PD initiation were obtained from electronic medical records. Patient survival was censored at kidney transplantation or at the date of last follow-up.

### Statistical analysis

All statistical analyses except for competing risk analysis were conducted using software (BellCurve for Excel; Social Survey Research Information Co., Ltd., Tokyo, Japan). Continuous data were expressed as the mean ± standard deviation or median (interquartile range). Student *t*-tests or Mann–Whitney U-tests were used to compare continuous variables between two groups. A Fisher’s exact test or chi-square test was used to compare categorical variables. Receiver operating characteristic (ROC) analysis was performed to evaluate the predictive ability of continuous variables for primary outcomes. The Kaplan–Meier method and the log-rank test were used to compare differences in primary outcomes between the groups. Univariate and multivariate Cox proportional hazards regression analyses were conducted to assess whether a target factor is associated significantly with the outcome. Competing risk analysis was performed using EZR (Jichi Medical University, Tochigi, Japan), which is a graphical user interface for R (The R Foundation for Statistical Computing, Vienna, Austria) [18]. Results for which a* p* value less than 0.05 were obtained were inferred as representing significant difference.

## Results

### Study participant characteristics

**Fig. 1:**
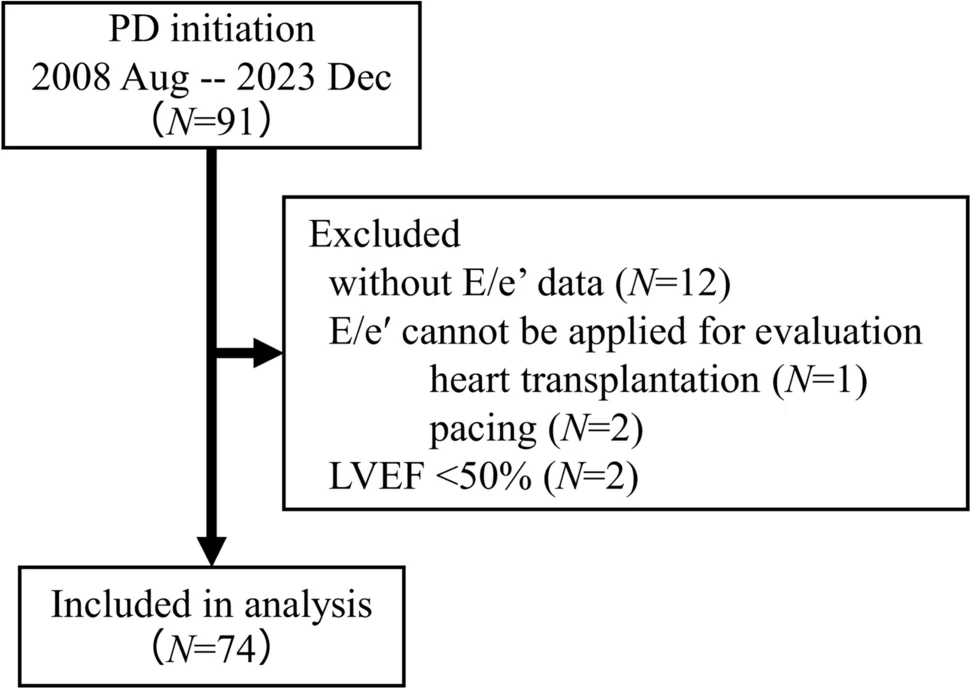
Flow diagram of inclusion and exclusion of the study participants

Of the 91 patients who started PD during the study period, 74 patients were included in the analyses (Fig. 1). Table 1 presents the patient characteristics and laboratory data. The median (IQR) of age was 64 (54, 71) years old; 55 (74%) were men. The median duration from PD initiation to echocardiographic examination was 3.3 (2.9, 3.8) months. The median value of E/e′ was 9.4 (7.5, 12.0).

**Table 1:** Baseline patient characteristics

Variable	All (*N* = 74)	E/e′ Avg ≤ 9.0 (*N* = 35)	E/e′ Avg > 9.0 (*N* = 39)	*p*
Age at PD initiation [y.o.]	64 (54, 71)	57 (51, 68)	69 (60, 73)	< 0.01
Male gender [*n* (%)]	55 (74%)	28 (80%)	27 (69%)	0.42
*Cause of kidney failure [n (%)]*				
Diabetic kidney disease	17 (23%)	5 (14%)	12 (31%)	0.18
Nephrosclerosis	12 (16%)	5 (14%)	7 (18%)	
Chronic glomerulonephritis	31 (42%)	19 (54%)	12 (31%)	
Others/unknown	14 (19%)	6 (17%)	8 (21%)	
CVD history [*n* (%)]	16 (22%)	6 (17%)	10 (26%)	0.41
ACE-i/ARB [*n* (%)]	59 (80%)	27 (77%)	32 (82%)	0.77
Beta blocker [*n* (%)]	18 (24%)	7 (20%)	11 (28%)	0.43
Body mass index [kg/m^2^]	23.2 ± 4.2	23.0 ± 3.8	23.4 ± 4.6	0.64
Systolic blood pressure [mmHg]	137 ± 17	138 ± 17	136 ± 17	0.54
Diastolic blood pressure [mmHg]	78 ± 13	82 ± 12	74 ± 14	0.01
Automated PD [*n* (%)]	58 (78%)	30 (86%)	28 (72%)	0.17
2.5% glucose solution use [*n* (%)]	6 (8%)	4 (11%)	2 (5%)	0.41
Icodextrin solution use [*n* (%)]	4 (5%)	1 (3%)	3 (8%)	0.62
Total weekly Kt/V	2.2 (1.9, 2.5)	2.1 (1.9, 2.4)	2.3 (2.0, 2.5)	0.20
Renal weekly Kt/V	1.2 (0.9, 1.5)	1.1 (0.8, 1.5)	1.3 (0.9, 1.6)	0.33
D/P Cr	0.56 (0.52, 0.66)	0.54 (0.50, 0.61)	0.61 (0.53, 0.67)	0.02
Hb [g/dl]	11.3 ± 1.0	11.2 ± 1.1	11.4 ± 0.9	0.64
Alb [g/dl]	3.6 ± 0.4	3.7 ± 0.4	3.5 ± 0.4	0.11
BUN [mg/dl]	52.6 ± 10.6	53.3 ± 11.2	52.1 ± 10.2	0.63
Cr [mg/dl]	6.7 (5.6, 8.1)	8.1 (6.0, 9.2)	6.5 (5.2, 7.2)	< 0.01
Corrected Ca [mg/dl]	9.1 (8.7, 9.4)	9.1 (8.8, 9.3)	9.1 (8.6, 9.4)	0.71
IP [mg/dl]	4.8 ± 0.8	5.0 ± 0.9	4.6 ± 0.8	0.07
CRP [mg/dl]	0.07 (0.03, 0.17)	0.07 (0.03, 0.20)	0.09 (0.04, 0.17)	0.74
BNP [pg/ml]	61.7 (27.2, 114.3)	43.2 (22.2, 70.3)	86.7 (47.8, 163.9)	< 0.01
LVEF [%]	67 (62, 71)	66 (63, 72)	67 (62, 71)	0.90
E/A	0.73 (0.54, 0.88)	0.78 (0.53, 1.07)	0.70 (0.56, 0.84)	0.47
E/e′ Avg	9.4 (7.5, 12.0)	7.4 (6.0, 8.5)	11.9 (10.6, 14.9)	< 0.01
LVH [*n* (%)]	16 (22%)	3 (9%)	13 (33%)	0.01

Continuous data are presented as mean ± SD or median (IQR): *PD* peritoneal dialysis; *CVD* cardiovascular disease; *ACE-i* angiotensin-converting enzyme inhibitor; *ARB* angiotensin II receptor blocker; *D/P Cr* dialysate-to-plasma creatinine ratio at 4h; *Hb* hemoglobin; *Alb* albumin; *BUN* blood urea nitrogen; *Cr* creatinine; *Ca* calcium; *IP* phosphate; *CRP* C-reactive protein; *BNP* brain natriuretic peptide; *LVEF* left ventricular ejection fraction; *E/e′ Avg* average of septal and lateral E/e′; *LVH* left ventricular hypertrophy

During the median follow-up period of 4.2 years, primary outcomes were recorded for 36 (49%) patients, of whom 6 died and 30 discontinued PD. The six patients died because of CVD (*n* = 4), infection (*n* = 1), and malignancy (*n* = 1). The causes of PD discontinuation included peritonitis (*n* = 11), exit-site and tunnel infection (*n* = 5), fluid overload (*n* = 4), short of solute removal (*n* = 3), and others (*n* = 7).

### Relation between E/e′ Avg and outcome

**Fig. 2:**
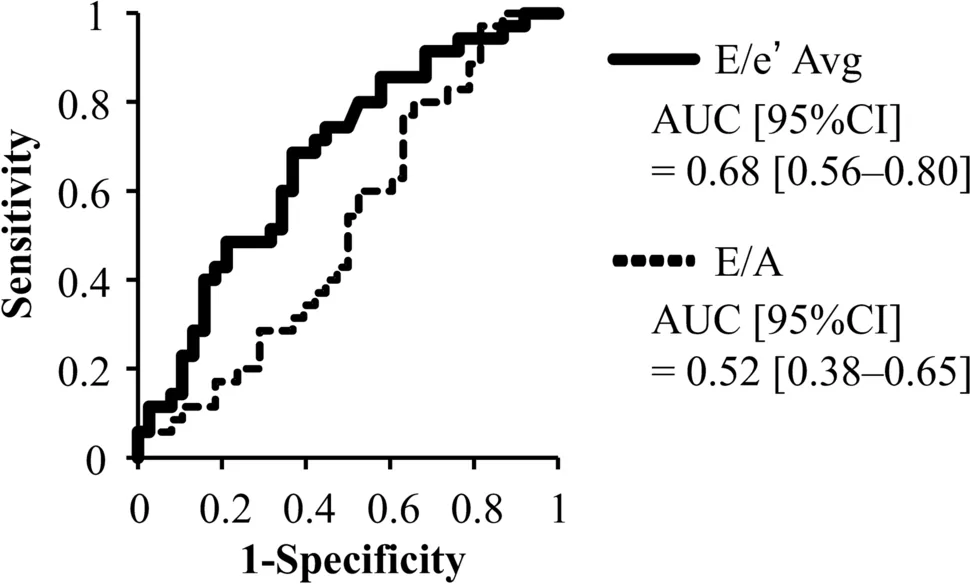
Receiver operating characteristic (ROC) analysis assessing the predictive ability of E/e′ Avg and E/A for 8-year mortality or PD discontinuation after starting PD

Subsequent ROC analysis to elucidate the predictive ability of E/e′ Avg or E/A for primary outcome showed that E/e′ Avg had moderate accuracy with area under the curve (AUC) [95% confidence interval (95% CI)] of 0.68 [0.56–0.80] and suggested the cut-off value of 9.05, but E/A did not (Fig. 2). Therefore, all patients were divided into two groups based E/e′ Avg less than or equal to 9.0 (*n* = 35) and higher than 9.0 (*n* = 39) (Table 1). The group with E/e′ Avg > 9.0 had significantly higher age [69 (60, 73) vs. 57 (51, 68) years old, *p* < 0.01], D/P Cr [0.61 (0.53, 0.67) vs. 0.54 (0.50, 0.61), *p* = 0.02], BNP [86.7 (47.8, 163.9) vs. 43.2 (22.2, 70.3) pg/ml, *p* < 0.01], and presence of LVH (33% vs. 9%, *p* = 0.01) than the group with E/e′ Avg ≤ 9.0. By contrast, diastolic blood pressure (74 ± 14 vs. 21 ± 12 mmHg, *p* = 0.03) and Cr [6.5 (5.2, 7.2) vs. 8.1 (6.0, 9.2) mg/dl, *p* < 0.01] were significantly lower in the group with E/e′ Avg > 9.0. The occurrence of composite outcomes was significantly higher in the group with E/e′ Avg > 9.0 than that with E/e′ Avg ≤ 9.0 (64% vs. 31%, *p* < 0.01), but the causes of PD discontinuation or death were not significantly different between the two groups (*p* = 0.11). No difference was observed in the E/e′ Avg between patients who discontinued participation because of PD-related infections and those who discontinued for other reasons (10.4 [8.7, 13.2] vs. 10.8 [8.5, 12.6], *p* = 0.90).

**Fig. 3:**
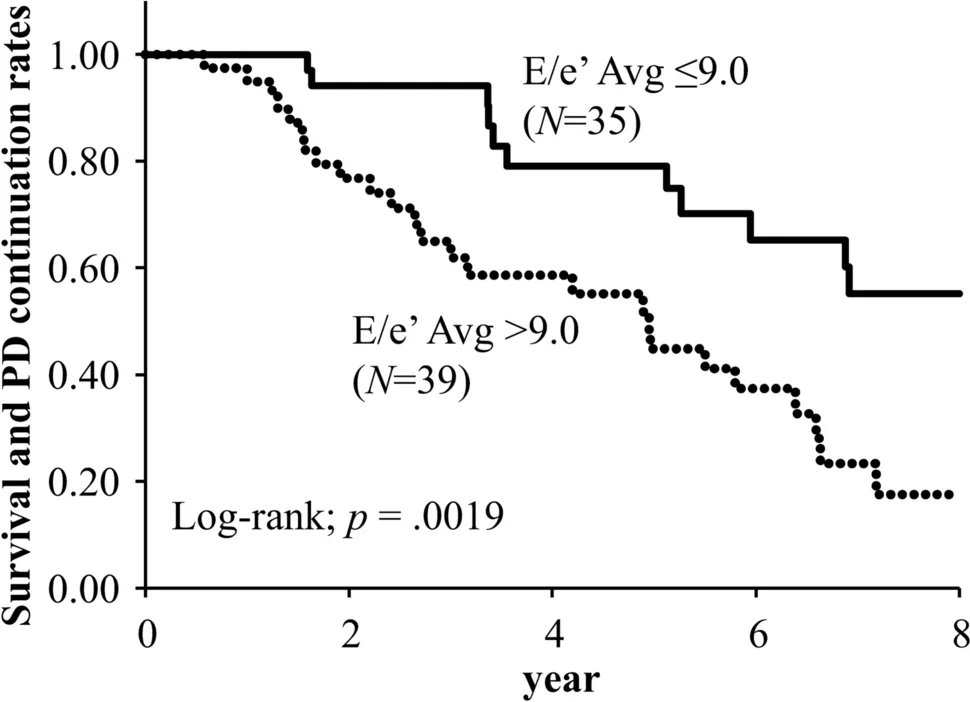
Kaplan–Meier analysis for primary outcome (death or PD discontinuation) within 8 years after PD initiation

Using Kaplan–Meier analysis and log-rank testing, the composite outcome of 8-year mortality or PD discontinuation was compared between the groups divided by E/e′ Avg (Fig. 3). Results indicated that the group with E/e′ Avg > 9.0 had significantly lower survival or PD continuation rate (*p* = *0.0*019). When mortality and PD discontinuation are evaluated separately, results from Gray test showed that the cumulative probability of death in the E/e′ Avg > 9.0 group was not significantly different from that in the E/e′ Avg ≤ 9.0 group, likely because of the small number of observed events (*p* = 0.121). However, the cumulative probability of PD discontinuation in the E/e′ Avg > 9.0 group was significantly higher than that in the E/e′ Avg ≤ 9.0 group (*p* = 0.0265) (Fig. S1). No significant difference was found in cessation of PD monotherapy between the two groups (*p* = 0.364) (Fig. S2).

Relations between primary outcome and clinical variables were evaluated using univariate and multivariate Cox proportional hazards model analyses (Table S1, Table 2). Results of univariate analysis suggested significant relations between outcome and Alb (hazards ratio (HR) [95%CI] = 0.36 [0.17–0.78], *p* < 0.01), BNP (HR [95%CI] = 1.00 [1.00–1.00], *p* < 0.01), use of icodextrin-based PD solution (HR [95%CI] = 9.55 [2.51–36.37], *p* < 0.01), and E/e′ Avg (HR [95%CI] = 1.15 [1.07–1.24], *p* < 0.01) (Table S1). Results of multivariate Cox analysis suggest that E/e′ Avg, Alb, and icodextrin solution use are associated significantly with the primary outcome (Table 2).

**Table 2:** Results from multivariate Cox proportional hazards model for death or PD discontinuation within 8 years

Variable	HR (95% CI)	*p*
*Model 1*		
E/e′ Avg	1.17 (1.07–1.28)	< 0.01
Age at PD initiation	1.00 (0.97–1.04)	0.99
Alb	0.35 (0.15–0.85)	0.02
Cr	1.09 (0.89–1.33)	0.39
*Model 2*		
E/e′ Avg	1.14 (1.05–1.24)	< 0.01
Age at PD initiation	0.99 (0.96–1.03)	0.74
Alb	0.37 (0.15–0.91)	0.03
BNP	1.00 (1.00–1.00)	0.08
*Model 3*		
E/e′ Avg	1.14 (1.05–1.24)	< 0.01
Age at PD initiation	1.01 (0.97–1.04)	0.71
BNP	1.00 (1.00–1.00)	0.051
LVH	0.88 (0.38–2.05)	0.76
*Model 4*		
E/e′ Avg	1.16 (1.07–1.26)	< 0.01
Age at PD initiation	0.99 (0.96–1.03)	0.65
Alb	0.51 (0.20–1.31)	0.02
Icodextrin solution use	6.47 (1.34–31.34)	0.02

*PD* peritoneal dialysis; *Alb* albumin; *Cr* creatinine; *BNP* brain natriuretic peptide; *E/e′ Avg* average of septal and lateral E/e′; *LVH* left ventricular hypertrophy

Generally, in patients with preserved LVEF, the upper limit of normal for the E/e′ ratio is regarded as 8. A value exceeding 15 is accepted as reflecting abnormal left ventricle relaxation and increased LV stiffness [19–21]. Therefore, sensitivity analysis was conducted by comparing outcomes between the two groups divided based on the value of E/e′ Avg of 15 or 8 (Fig. S3). Results obtained from Kaplan–Meier analysis suggest that the patient groups with E/e′ Avg > 15 and E/e′ Avg > 8 had significantly higher mortality and PD discontinuation rates, respectively, than those with E/e′ Avg ≤ 15 (*p* = *0.0*065) and E/e′ Avg ≤ 8 (*p* = 0.0158).

## Discussion

Our study demonstrated that increased E/e′ Avg soon after PD initiation is associated significantly with each of 8-year mortality and PD discontinuation. A cut-off value of E/e′ Avg > 9.0 is inferred as predictive of adverse outcomes.

A retrospective study including maintenance PD patients demonstrated that patients with E/e′ ≥ 15 had significantly worse survival and a higher risk of heart failure than patients with E/e′ < 15 [6]. Results from another study showed that LVDD defined as E/e′ > 15 is a significant risk factor of CV mortality in kidney failure patients on HD or PD with preserved LV systolic function [14]. For incident dialysis patients with preserved LV systolic function, E/e′ > 15 or left atrial volume index (LAVI) > 32 mL/m^2^ was associated significantly with CV events. However, relations between these indices of LVDD and adverse events were diminished in a subgroup analysis of PD patients [7]. A longitudinal observational study including incident PD patients demonstrated E/e′ > 15 as an independent predictor for loss of residual kidney function and cardiac events [8].

Although the exact mechanisms by which the E/e′ ratio is associated with prognosis in PD patients remain unclear, consideration of factors affecting E/e′ in dialysis patients might provide some explanations. Among the E/e′ components, increased velocity of E-wave can be caused mainly by fluid overload [4, 22]. In fact, fluid overload is known as a common risk factor of death or transfer to HD in PD patients [2, 23, 24]. The LVH, as represented by elevated LVMI, is also one of the characteristic causes of increased E/e′ in kidney failure patients [6, 25]. In PD patients, LVH is a risk factor for adverse outcomes including mortality, PD discontinuation, and CV events [16, 26]. In our study, results obtained from comparison of two groups divided by E/e′ Avg showed BNP and prevalence of LVH as significantly higher in the higher E/e′ Avg group than in the lower E/e′ Avg group. E/e′ might serve as an integrated marker reflecting the influences of multiple factors including volume overload and structural changes. The relation between E/e′ and infection, which is the most common cause of PD discontinuation, has not been fully clarified. However, considering the fact that elevated E/e′ correlates with mortality in septic patients, increased E/e′ might contribute to worsening clinical outcomes following infection [27]. Increased E/e′ is reportedly associated with all-cause mortality in both HD and PD patients, in whom infection is one common cause of death [6, 10].

Regarding the definition of LVDD, several studies involving PD patients have set the cut-off value for E/e′ as higher than 15, which is generally accepted as reflecting abnormal left ventricle relaxation and increased LV stiffness in patients with preserved LVEF [6–8, 14, 19, 20]. Indeed, results from the present study show that patients with E/e′ Avg higher than 15 had significantly higher mortality and PD discontinuation rates than those with E/e′ Avg less than or equal to 15 (Fig. S3). However, findings from this study suggest an optimal cut-off value of > 9.0 for E/e′ Avg (much lower than 15) to predict mortality and PD discontinuation. Results of our study and of other studies suggest different cut-off values, perhaps because of differences in the distribution of E/e′ values within the respective study cohorts. The median value of E/e′ Avg found from our study was 9.4, whereas the mean values of E/e′ found from earlier studies were 13.0–15.73 [6–8]. Differences in E/e′ distributions between patient cohorts might be attributable to differences in clinical factors affecting E/e′, such as LVH. Prevalence of LVH was 23% in our study, whereas more than half of participants had LVH in earlier studies [6, 8]. The cut-off value proposed in this study was higher than 8, which is the generally accepted upper limit of the normal range [21].

A mild to moderate increase in the E/e′ ratio is regarded as a “gray zone” in which left ventricular filling pressure cannot be ascertained based on this value alone [3]. In our study, when patients with E/e′ Avg > 9.0 were divided into two groups, those with values between 9.0 to 15.0 (*N* = 29) and those with values above 15.0 (*N* = 10), the incidence of the primary outcome was comparable (Log-rank, *p* = 0.144), and there were no differences in patient baseline parameters except for BNP (84.4 [30.4, 115.5] vs. 193.1 [88.1, 404.3] pg/ml, *p* = 0.02). These findings might indicate that even patients with intermediate increases in the E/e′ ratio are at risk of PD discontinuation or death, suggesting the need for careful follow-up. They also suggest that the value of E/e′ Avg reflects not only LVDD but also left ventricular volume load or myocardial damage in our PD patients.

The E/e′ ratio is recognized as a reliable and sensitive marker of LVDD in patients undergoing kidney replacement therapy [7, 12, 13]. For patients on PD, indices of left ventricular diastolic function including the E/e′ ratio changed considerably during three years, whereas indices of LV structure such as LVMI did not, indicating that the progressions of functional and structural changes were not parallel [15]. Studies including PD patients showed E/e′ as associated significantly with mortality or CV events, whereas LVMI was not [6, 8]. These findings suggest the clinical significance and importance of evaluating E/e′ in patients on PD.

Because the E/e′ ratio can become higher under the influence of multiple clinical factors including long dialysis vintage and fluid overload, interpretation of E/e′ might require consideration of the timing and patient condition [6, 22]. In our study, echocardiographic examination was performed early after PD initiation. In fact, all cases were conducted 2–6 months after starting PD, with a median of 3.3 months. By this time, a PD prescription tailored to the patient would be established and residual kidney renal function could be preserved, making it easier to stabilize fluid management. Therefore, the timing of echocardiographic examination as part of our routine treatment for PD patients might be appropriate for baseline E/e′ assessment.

It has been proposed that chronic low-grade systemic inflammation triggered by underlying conditions or diseases is one of mechanisms of the development of LVDD [28, 29]. Under several conditions or systemic diseases such as aging, obesity, hypertension, diabetes mellitus, and chronic kidney disease, a systemic inflammatory state is induced and pro-inflammatory cytokines are released by multiple pathways such as immune reaction, cell hypoxemia, activation of neuroendocrine, and autonomic nerve function disorder. A prolonged systemic proinflammatory state causes microvascular endothelial dysfunction, which might engender structural and functional deterioration of cardiomyocyte and cardiac interstitial fibrosis. An increased E/e′ might, in part, reflect the accumulation of chronic systemic inflammation caused by systemic diseases and patient states that elevate the risk of cardiovascular disease or death.

Results obtained from this study indicated that a lower serum albumin concentration is associated with increased risk of death and PD discontinuation. Earlier reports have described that hypoalbuminemia at the PD initiation period is associated with adverse outcomes including mortality, peritonitis within 6 months, and transfer from PD to HD [30–32]. Several possible reasons why serum albumin is associated with PD continuation and survival have been proposed. Serum albumin might be regarded as a reactive index of overall health; its concentration decreases in response to various conditions, such as malnutrition, inflammation, fluid overload, and excessive protein loss to dialysate, all of which adversely affect a PD patient’s prognosis. Moreover, serum albumin contributes to the maintenance of colloid osmotic pressure, and a decrease in its concentration can engender pulmonary congestion. Because serum albumin is involved in the process of antioxidant, damage repair, and immune response, a decrease in its blood concentration might impair the body’s protective functions against diseases [30, 31].

Our results showed the positive correlation between icodextrin-based PD solution use and the occurrence of primary outcome. Given our clinical policy of using icodextrin in cases where fluid management is inadequate with glucose-based dialysate alone, the results presented above suggest that fluid overload early after PD initiation is a possible risk for adverse outcomes, death or PD discontinuation.

Our study had several limitations. First, because this study was conducted at a single center with a small number of patients, it remains unclear whether the results are applicable to PD patients in other hospitals. Second, E/e′ Avg was evaluated only at baseline in this study. A future investigation must address longitudinal changes of E/e′ Avg and their association with outcomes. Third, other indices of LVDD such as tricuspid regurgitant velocity and LAVI could not be evaluated because of numerous missing data. Although E/e′ has benefits in terms of its feasibility and reproducibility, other echocardiographic parameters including LAVI and left atrial strain are reportedly associated with mortality or major cardiovascular events in patients undergoing PD [33, 34]. Further research must be conducted to achieve more accurate risk stratification using multiple LVDD indices. To address these limitations, a large, prospective observational study should be conducted.

In conclusion, this study demonstrated that the E/e′ ratio evaluated soon after PD initiation is independently associated with 8-year mortality and PD discontinuation in PD patients with preserved LVEF. The baseline E/e′ ratio can be a prognostic marker that might contribute to risk stratification and treatment planning in patients starting PD. Additional large studies must be conducted to reinforce the results obtained from this study.

## Supplementary Information

Below is the link to the electronic supplementary material.

Supplementary file1 (PPTX 103 KB)

Supplementary file2 (DOCX 34 KB)

## Data Availability

The data which support the findings of this study are available from the corresponding author Yoshifumi Hamasaki upon reasonable request.
