# Supplementary material for: An index of left ventricular diastolic dysfunction in patients starting peritoneal dialysis might indicate mortality and peritoneal dialysis discontinuation
Source: Clin Exp Nephrol. 2026 Jul 11;30(11):1598–606. doi: 10.1007/s10157-026-02913-x (PMC13669133; doi:10.1007/s10157-026-02913-x)
Supplement: Supplementary file 1 [file 10157_2026_2913_MOESM2_ESM.docx]

Figure S1. Competing risk analysis for death (Fig. S1a) and PD discontinuation (Fig. S2a) within 8 years after PD initiation

Figure S2. Kaplan–Meier analysis for cessation of PD monotherapy within 8 years after PD initiation

Figure S3. Sensitivity analysis comparing two groups divided based on E/e′ Avg using Kaplan–Meier method and log-rank testing

Table S1. Results of univariate Cox proportional hazards model analyses for death or PD discontinuation within 8 years

| **Variable** | **HR (95% CI)** | ***p*** |
| --- | --- | --- |
| age | 1.02 (0.99–1.05) | .16 |
| male gender | 0.74 (0.35–1.58) | .44 |
| diabetes history | 1.78 (0.87–3.62) | .11 |
| CVD history | 0.96 (0.42–2.19) | .92 |
| ACE-i/ARB | 0.85 (0.37–1.94) | .69 |
| beta blocker | 1.87 (0.91–3.83) | .09 |
| body mass index | 1.08 (0.99–1.18) | .09 |
| systolic blood pressure | 0.99 (0.97–1.01) | .56 |
| diastolic blood pressure | 0.98 (0.95–1.00) | .07 |
| 2.5% glucose solution use | 0.97 (0.23–4.10) | .97 |
| icodextrin solution use | 9.55 (2.51–36.37) | < .01 |
| automated PD | 1.07 (0.47–2.45) | .87 |
| total weekly Kt/V | 1.12 (0.54–2.36) | .76 |
| renal weekly Kt/V | 1.39 (0.71–2.72) | .33 |
| D/P Cr | 3.05 (0.14–65.02) | .48 |
| Hb | 0.85 (0.60–1.20) | .36 |
| Alb | 0.36 (0.17–0.78) | < .01 |
| BUN | 1.00 (0.97–1.03) | .82 |
| Cr | 0.94 (0.79–1.12) | .49 |
| corrected Ca | 1.34 (0.73–2.48) | .35 |
| IP | 1.08 (0.72–1.63) | .71 |
| CRP | 1.10 (0.81–1.49) | .53 |
| BNP | 1.00 (1.00–1.00) | < .01 |
| LVEF | 1.01 (0.96–1.05) | .81 |
| E/A | 0.53 (0.14–1.99) | .35 |
| E/e’ Avg | 1.15 (1.07–1.24) | < .01 |
| LVH | 1.52 (0.71–3.24) | .28 |

PD, peritoneal dialysis; CVD, cardiovascular disease; ACE-i, angiotensin-converting enzyme inhibitor; ARB, angiotensin II receptor blocker; D/P Cr, dialysate-to-plasma creatinine ratio at 4 hr; Hb, hemoglobin; Alb, albumin; BUN, blood urea nitrogen; Cr, creatinine; Ca, calcium; IP, phosphate; CRP, C-reactive protein; BNP, brain natriuretic peptide; LVEF, left ventricular ejection fraction; E/e′ Avg, average of septal and lateral E/e′; LVH, left ventricular hypertrophy
